# Mycophenolate Mofetil Treatment Reduces the Risk of Treatment Escalation Due to Vascular Complications in Limited Cutaneous Systemic Sclerosis: Emulation of a Target Trial From the Italian Rheumatology Society SPRING Registry

**DOI:** 10.1002/acr.70039

**Published:** 2026-04-17

**Authors:** Enrico De Lorenzis, Gerlando Natalello, Rossella De Angelis, Lucrezia Verardi, Dilia Giuggioli, Gianluigi Bajocchi, Lorenzo Dagna, Silvia Bellando‐Randone, Giovanni Zanframundo, Rosario Foti, Fabio Cacciapaglia, Giovanna Cuomo, Alarico Ariani, Edoardo Rosato, Gemma Lepri, Francesco Girelli, Valeria Riccieri, Elisabetta Zanatta, Ilaria Cavazzana, Francesca Ingegnoli, Maria De Santis, Giuseppe Murdaca, Giuseppina Abignano, Giorgio Pettiti, Alessandra Della Rossa, Maurizio Caminiti, Annamaria Iuliano, Giovanni Ciano, Lorenzo Beretta, Gianluca Bagnato, Ennio Lubrano, Maria Ilenia De Andres, Alessandro Giollo, Cosimo Bruni, Martina Orlandi, Marco Fornaro, Marta Saracco, Cecilia Agnes, Pier Giacomo Cerasuolo, Gabriella Alonzi, Edoardo Cipolletta, Federica Lumetti, Amelia Spinella, Luca Magnani, Corrado Campochiaro, Giacomo De Luca, Veronica Codullo, Elisa Visalli, Carlo Iandoli, Antonietta Gigante, Greta Pellegrino, Erika Pigatto, Maria‐Grazia Lazzaroni, Franco Franceschini, Elena Generali, Gianna Mennillo, Simone Barsotti, Giuseppa Pagano Mariano, Federica Furini, Licia Vultaggio, Simone Parisi, Clara Lisa Peroni, Gerolamo Bianchi, Enrico Fusaro, Gian Domenico Sebastiani, Marcello Govoni, Salvatore D'Angelo, Franco Cozzi, Fabrizio Conti, Serena Guiducci, Andrea Doria, Carlo Salvarani, Florenzo Iannone, Maria Antonietta D'Agostino, Clodoveo Ferri, Marco Matucci Cerinic, Silvia Laura Bosello, the SPRING Italian Registry Collaborators

**Affiliations:** ^1^ Division of Rheumatology and Clinical Immunology Fondazione Policlinico Universitario A. Gemelli IRCCS Rome Italy; ^2^ Rheumatology, Faculty of Medicine and Surgery Catholic University of the Sacred Heart Rome Italy; ^3^ Rheumatology Unit, Department of Clinical and Molecular Sciences Polytechnic University of Marche Ancona Italy; ^4^ Department of Medical and Surgical Sciences for Children and Adults University Hospital of Modena and Reggio Emilia School of Medicine Modena Italy; ^5^ Rheumatology Unit Azienda USL IRCCS di Reggio Emilia Reggio Emilia Italy; ^6^ Unit of Immunology, Rheumatology, Allergy and Rare Diseases, IRCCS San Raffaele Scientific Institute Vita‐Salute San Raffaele University Milan Italy; ^7^ Division of Rheumatology, Scleroderma Unit, Department of Experimental and Clinical Medicine, AOU Careggi University of Florence Florence Italy; ^8^ Department of Internal Medicine and Therapeutics Università di Pavia Pavia Italy; ^9^ Division of Rheumatology Fondazione IRCCS Policlinico San Matteo Pavia Italy; ^10^ Rheumatology Unit AOU Policlinico S. Marco Catania Italy; ^11^ Rheumatology Service “F. Miulli” General Hospital Acquaviva delle Fonti ‐ Department of Medicine and Surgery LUM University “Giuseppe De Gennaro” Casamassima Bari Italy; ^12^ Department of Precision Medicine University of Campania Luigi Vanvitelli Caserta Italy; ^13^ Department of Medicine, Internal Medicine and Rheumatology Azienda Ospedaliero Universitaria di Parma Parma Italy; ^14^ Department of Translational and Precision Medicine Sapienza University of Rome Roma Italy; ^15^ Rheumatology Unit, AUSL Romagna Ospedale G.B. Morgagni Forlì Italy; ^16^ Department of Rheumatology Sapienza University of Rome Rome Italy; ^17^ Department of Rheumatology University of Padua Padua Italy; ^18^ Department of Cardiac, Thoracic, Vascular Sciences and Public Health University of Padua Padua Italy; ^19^ Rheumatology and Clinical Immunology ASST Spedali Civili of Brescia; Department of Clinical and Experimental Sciences University of Brescia Brescia Italy; ^20^ Rheumatology Clinic, ASST Pini CTO, Department of Clinical Sciences & Community Health Università degli Studi di Milano Milan Italy; ^21^ Rheumatology and Clinical Immunology IRCCS Humanitas Research Hospital Rozzano Milan Italy; ^22^ Department of Biomedical Sciences Humanitas University Pieve Emanuele Milan Italy; ^23^ Research Hospital San Martino University of Genoa Genoa Italy; ^24^ Rheumatology Institute of Lucania (IReL) and Rheumatology Department of Lucania San Carlo Hospital Potenza Italy; ^25^ Azienda Ospedaliera S Croce e Carle Cuneo Italy; ^26^ Department of Rheumatology University of Pisa Pisa Italy; ^27^ Departmental Rheumatology Unit Grande Ospedale Metropolitano Reggio Calabria Italy; ^28^ Rheumatology Unit San Camillo ‐ Forlanini Hospital Rome Italy; ^29^ Hospital of Ariano Irpino, Local Health Department Ariano Irpino Avellino Italy; ^30^ Referral Center for Systemic Autoimmune Diseases Fondazione IRCCS Ca' Granda, Ospedale Maggiore Policlinico di Milano Milan Italy; ^31^ Department of Clinical and Experimental Medicine University of Messina Messina Italy; ^32^ Department of Rheumatology University of Molise Campobasso Italy; ^33^ Rheumatology Unit Azienda Ospedaliera di Rilievo Nazionale e di Alta Specializzazione “Garibaldi,” Catania Italy; ^34^ Rheumatology Section, Department of Medicine University of Verona Verona Italy; ^35^ Rheumatology Unit, Department of Precision and Regenerative Medicine Ionian Area University of Bari Aldo Moro Bari Italy; ^36^ Rheumatology Unit Mauriziano‐Umberto I Hospital Torino Italy; ^37^ San Lorenzo Hospital Carmagnola Turin Italy; ^38^ IRCCS Ospedale Galeazzi Sant'Ambrogio Milan Italy; ^39^ Dipartimento di Scienze Biomediche e Cliniche Università degli Studi di Milano Milan Italy; ^40^ Medicine Unit, Department of Medicine San Bassiano Hospital Bassano del Grappa Italy; ^41^ Rheumatology Unit, Department of Medical Sciences University of Ferrara and Azienda Ospedaliera‐Universitaria S. Anna di Ferrara Ferrara Italy; ^42^ Rheumatology Unit University Hospital Città della Salute e della Scienza di Torino Turin Italy; ^43^ Rheumatology Unit, Department of Musculoskeletal Sciences Local Health Trust La Colletta Hospital Genoa Italy; ^44^ Department of Health Science University of Basilicata Potenza Italy; ^45^ Rheumatology Unit, Azienda USL IRCCS di Reggio Emilia University of Modena and Reggio Emilia Reggio Emilia Italy; ^46^ Inflammation Fibrosis and Ageing Initiative (INFLAGE), Division of Genetics and Cell Biology IRCCS San Raffaele Scientific Institute Milan Italy

## Abstract

**Objective:**

Mycophenolate mofetil (MMF) use in limited cutaneous systemic sclerosis (lcSSc) is relatively uncommon because of the lower fibrotic burden and the predominance of vascular complications. In vitro observations and clinical data from transplanted patients suggest a protective effect of MMF on endothelial function. Our aim was to evaluate the reasons for prescribing MMF treatment in patients with lcSSc and its impact on the need for escalation of vascular complication–related treatments during follow‐up.

**Methods:**

Patients with lcSSc enrolled in the Italian Systemic Sclerosis Progression Investigation registry were retrospectively evaluated. All patients treated with MMF were matched to patients not treated with MMF, which was based on a roll‐entry time‐dependent propensity score built on demographics, clinical features, and baseline treatment. The escalation of vasoactive or vasodilator treatment up to 60 months was defined as the introduction of iloprost, endothelin receptor antagonists, or phosphodiesterase‐5 inhibitors on top of the ongoing treatment, because of uncontrolled or newly diagnosed vascular complications. A hazards Cox model was also adopted to quantify the association of MMF treatment with treatment escalation.

**Results:**

A total of 1,435 patients with lcSSc were evaluated, of whom 152 were prescribed MMF (17.1% male; mean age at lcSSc onset 48.7 ± 13.9 years, 54.6% anti‐Scl70 positive). The prescription of MMF was more common in men and in anti‐Scl70 positive, anticentromere negative patients with interstitial lung disease, myositis, and without a history of digital ulcers. After matching 107 patients with MMF‐untreated controls, the overall incidence of vasoactive/vasodilator treatment escalation events related to digital ulcers over a median follow‐up of 40.5 months (interquartile range 23.3–60.0) was 0.3 per 100 patient‐years in the MMF‐treated group and 5.4 per 100 patient‐years in the matched control group, with a significant difference in treatment escalation‐free survival between the two groups (hazard ratio 0.05, 95% confidence interval 0.01–0.38; *P* value = 0.004).

**Conclusion:**

In patients with lcSSc, the introduction of MMF has reduced the need for escalation of vasoactive or vasodilator treatment, suggesting that it may also help to prevent vascular complications, which frequently affect patients with lcSSc.

## INTRODUCTION

Systemic sclerosis (SSc) is a chronic autoimmune disease characterized by vascular, immune, and fibrotic complications that can affect the skin, the musculoskeletal system, and the internal organs. The limited cutaneous SSc (lcSSc) subset is the most frequent disease form^1^ and accounts for three of four patients. It features a reduced skin extension and a less frequent or severe presentation of pulmonary and cardiac fibrosis. LcSSc, in particular, is associated with significant vascular manifestations such as Raynaud phenomenon, digital ulcers (DUs), and pulmonary arterial hypertension (PAH).^2^, ^3^ The sine‐scleroderma subset is close to the lcSSc subset because of the absence of skin involvement but it has similar vascular and visceral patterns that contribute to defining SSc as a vascular disease characterized by a unified vascular phenotype.^4^, ^5^

**SIGNIFICANCE & INNOVATIONS**

- Although mycophenolate mofetil (MMF) is increasingly used in diffuse cutaneous systemic sclerosis (dcSSc) for its antifibrotic and immunomodulatory properties, its use in limited cutaneous SSc (lcSSc) remains uncommon, primarily due to the lower fibrotic burden and predominance of vascular manifestations.
- The prescription of MMF in lcSSc seems to be linked to inflammatory or fibrotic complications.
- MMF markedly lowers the need for escalation of vasoactive or vasodilator therapies targetingvascular complications in lcSSc.
- Collectively, these findings challenge the current paradigm that limits MMF use to fibrotic SSc subsets and open new avenues for its use as a dual‐acting therapy targeting both inflammation/fibrosis and vasculopathy in SSc.

Based on available randomized controlled trials,^6^, ^7^ immunosuppression is only recommended for patients with SSc with established inflammatory and fibrotic complications because it demonstrates a better outcome in this subset as compared with placebo. As a result, patients with lcSSc are less frequently treated with these medications as compared with diffuse cutaneous SSc (dcSSc), for which these complications are far more common. This therapeutic approach seems counterintuitive, given that immune activation in SSc is extensively connected to microvascular impairment and fibrosis^8^ across disease subsets.

Among the available immunosuppressants, mycophenolate mofetil (MMF) is extensively used as first‐line treatment for SSc interstitial lung disease (ILD), and it remains an option for severe cutaneous involvement or SSc‐associated myositis. In animal models and in vitro, MMF has been shown to control some aspects of the pathogenesis of SSc vascular complications, including endothelial activation and vascular intimal proliferation.^9^, ^10^, ^11^, ^12^, ^13^ MMF has also been suggested to exert benefits on the microcirculation of transplanted patients when compared with alternative immunosuppressive regimens.^8^, ^14^, ^15^ Our aim was to investigate the reason for introducing MMF treatment and its impact on the escalation of vasoactive or vasodilatory treatment due to vascular complications in patients with a lcSSc phenotype who were enrolled in a large national cohort in the Italian Society of Rheumatology (SIR)–Systemic Sclerosis Progression INvestiGation (SPRING) registry. This study represents the first large, propensity score–matched analysis to explore a rationale for MMF impact on vascular outcomes in lcSSc. The results demonstrate a marked reduction in the need for escalation of vasoactive or vasodilator therapy among MMF‐treated patients, indicating a possible vascular‐protective role of MMF beyond its established activity.

## METHODS

### Study design and participants

The research design and the results report adhered to the Strengthening the Reporting of Observational Studies in Epidemiology (STROBE) initiative guidelines.^16^ The study involved a quasi‐experimental cohort comparison of patients exposed to MMF alongside a parallel control group not exposed to MMF, which were matched using a roll‐entry strategy based on a dynamic, time‐dependent propensity score.

The eligible patients were enrolled in the SPRING registry, a multicenter, nonprofit national cohort study initiated by the SIR. This registry, which focused on patients with SSc, involved a total of 38 national tertiary centers with specific expertise in managing the condition.^17^ The study protocol was approved by the local ethical committees of each participating center, and all patients provided written informed consent.

All patients included in the analysis met the American College of Rheumatology/EULAR 2013 classification criteria for SSc,^18^ were classified as lcSSc according to LeRoy et al criteria,^1^ and had available information regarding MMF exposure. Electronic health records were evaluated using a standard workflow specifically developed for the SPRING research protocol and fulfilled by the clinicians directly in charge of the patients with SSc.^19^

Data collection was conducted using REDCap electronic tools. Variables of interest included demographics, the Charlson Comorbidity Index (CCI) at the end of observation,^20^ age at SSc onset, disease duration, presence of anticentromere antibodies (ACA) and anti‐Scl70 antibodies, and disease domains involved. These domains included ILD, which was defined as parenchymal involvement over 10% on high‐resolution computed tomography (HRCT), presence of DUs, myositis, and cardiac involvement. The severity of lung involvement was assessed based on the most recent forced vital capacity (FVC) and the diffusing capacity of the lungs for carbon monoxide (DLco). For each previous and ongoing treatment, the start and end dates were recorded in the registry.

### Rationale of exposure and endpoint definitions

For the quasi‐experimental analysis, we designated three months as the minimum treatment duration for a patient to be considered exposed to MMF, considering the common practice of dose titration and the anticipated delay between initiating this medication and observing any clinical benefits for labeled MMF use. In the same analysis, we chose the escalation of vasodilator or vasoactive treatment as the endpoint. This escalation was defined as the introduction of intravenous iloprost, endothelin receptor antagonists, or phosphodiesterase type 5 inhibitors (PDE5i) to the existing treatment regimen due to uncontrolled or newly diagnosed vascular manifestations (eg, severe Raynaud phenomenon, DUs or pitting scars, and PAH). The initiation of calcium channel blockers (CCB) was not categorized as an event because of its established role as standard background therapy for most patients with SSc, irrespective of the development of vascular complications. Nevertheless, CCB was regarded as a potential confounder in statistical analysis.

The preference for therapeutic escalation as an endpoint over the formal diagnosis of new vascular complications was based on its expected robustness to minimize selection, information, recall, observer, and misclassification biases. This approach acknowledges the difficulties of retrospectively defining a worsening of acral vascular disease in a reliable way and the challenges in accessing right heart catheterization in real‐world settings.

### Statistical analysis

Categorical variables were presented as numbers and percentages, whereas continuous variables were reported as mean ± SD or median with interquartile range (IQR), depending on the normality of the data, which were assessed through the inspection of quantile‐quantile plots. Cross‐sectional comparisons between patient groups receiving MMF and those not receiving MMF were conducted using the chi‐square test or Fisher exact test for categorical variables and the Mann‐Whitney U test or *t*‐test for continuous variables, as appropriate.

**Figure 1:**
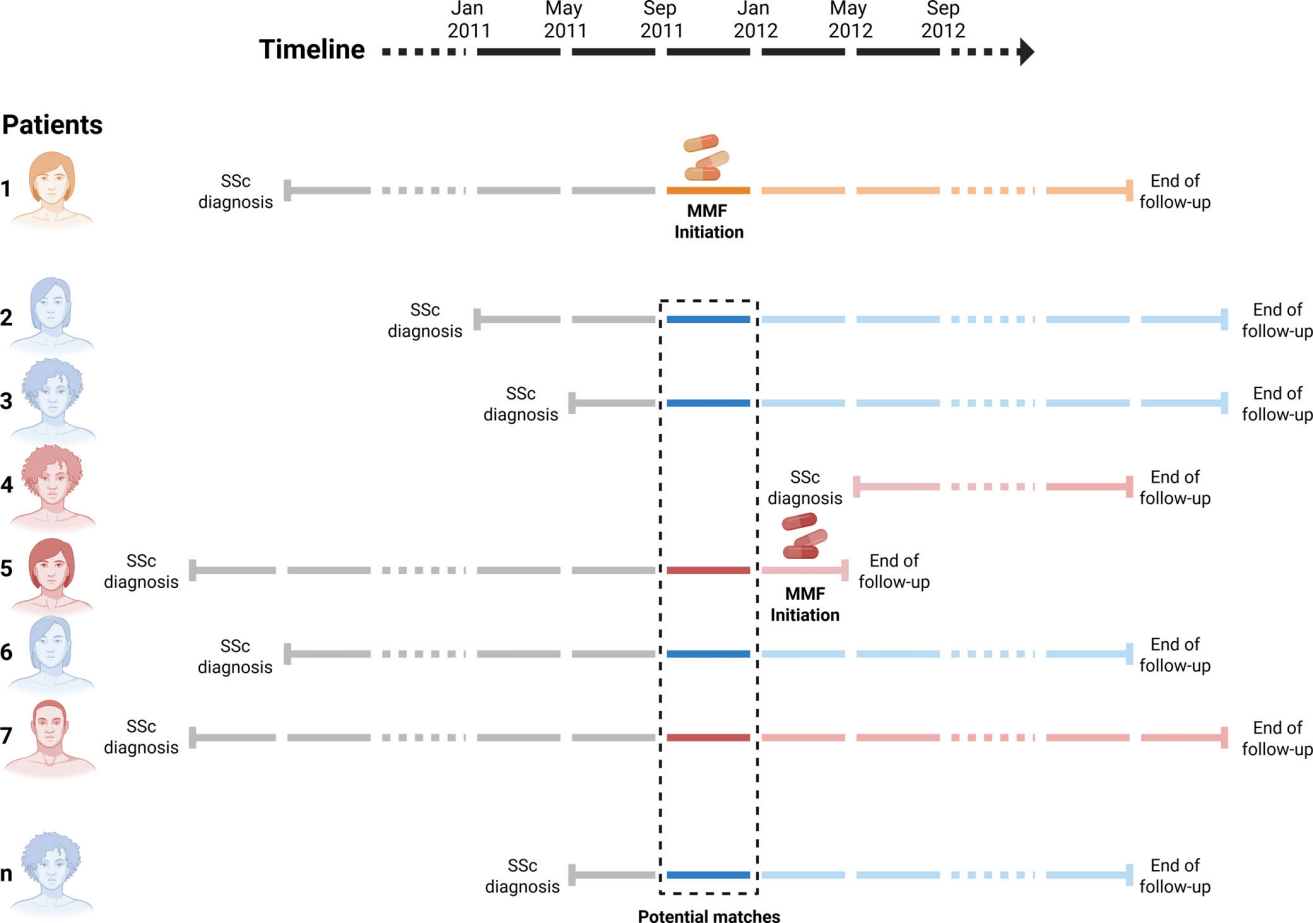
Example roll‐entry matching strategy: Patient 1 initiated MMF treatment in the four‐month period spanning September 2011 to January 2012. Patient 5, along with any other patient treated with MMF for at least three months, could not be matched. Similarly, patient 4, and any other patient diagnosed after that four‐month period or concluding the follow‐up before that four‐month period, could not be matched. The propensity score of patient 1 calculated in the same four‐month period was compared with that of the remaining patients. Patient 7, along with any other patients whose propensity score is too distant from that of patient 1 according to the established caliper, was excluded a priori. Finally, patient 1 was matched with patients among the remaining group with the closest propensity score calculated for that four‐month period. Color code: orange (MMF‐treated patients to be matched), blue (MMF‐untreated patients selected for matching), red (patients who could not be matched). Gray segments indicate disease intervals preceding the four‐month period of interest. Created with BioRender.com. MMF, mycophenolate mofetil; SSc, systemic sclerosis.

A roll‐entry strategy was adopted to select a matched control group.^21^ The duration of disease from diagnosis to the last follow‐up was divided into four‐month intervals for each patient. We selected four‐month intervals because this period reflects the average frequency of follow‐up assessments in clinical practice for patients with SSc, making it a clinically relevant timeframe for monitoring disease evolution. Moreover, this interval was expected to balance sufficient granularity for detecting clinically meaningful changes while avoiding excessive fragmentation of the data. A dynamic propensity score was calculated for each four‐month period using a binomial logistic model that incorporated both time‐independent and time‐dependent variables evaluated at the end of the previous four‐month period. Time‐dependent variables included age, disease duration from the first symptoms (excluding Raynaud phenomenon), and baseline treatment with CCBs, bosentan, macitentan, ambrisentan, sildenafil, tadalafil, rituximab, and tocilizumab, which were individually considered. Time‐independent variables included sex, anti‐Scl70 positivity, ACA positivity, and CCI. The last available FVC and DLco values were also approximated as time‐independent matching variables that served as general proxies for overall comorbidity burden and severity of lung involvement. We evaluated patients who were MMF‐exposed in the four‐month period of medication initiation and finally matched each of them with control patients who were among those evaluated in the same four‐month period along the timeline. Some patients were preliminarily excluded to prevent variable imbalance if their propensity scores differed significantly from the pooled propensity score of the treatment group. Patients were excluded if their propensity scores exceeded a prespecified fraction of the pooled propensity score SD that was defined by a caliper coefficient set at one SD of the pooled propensity score of the MMF‐treated group. The matching was one‐to‐one without replacement and based on the closest dynamic propensity score for that specific four‐month period (Figure 1). Standardized mean differences (SMDs) after matching for variables in the propensity score model were reported as a measure of balance between the intervention and comparison group with values 0.1 or below indicating a good balance.^22^

Kaplan‐Meier survival analysis was conducted to compare patients who were MMF‐exposed and the matched control group in preventing the endpoint based on the Log‐Rank test. Censoring rate and pattern similarity assumptions were assessed. A univariate proportional hazards Cox model was also adopted to quantify the association of MMF treatment and the endpoint given group balance of clinical variables. Results were presented as hazard ratios (HR) with 95% confidence intervals (CI). The proportional hazards assumption was verified by excluding statistically significant correlations between scaled Schoenfeld residuals and time. For both analyses, the observation period ended at the occurrence of an event, discontinuation of MMF for the treatment group, loss to follow‐up, or at the 60th month.

Statistical significance was defined as a *P* value less than 0.05 for all analyses, and all tests were two‐tailed. A Bonferroni correction was performed in case of multiple comparisons. Data analysis was performed using RStudio.

### Bias adjustment and missing data handling

Multiple bias mitigation strategies were adopted to enhance validity and reliability. First, uniform methods and tools for retrospective data collection across all study centers and patients were implemented to mitigate information bias according to the SPRING research protocol. Second, for the preliminary cross‐sectional comparison of patients receiving or not receiving MMF, a sensitivity analysis was conducted for patient subgroups diagnosed in three time periods: up to the publication of the 2009 EULAR systemic sclerosis guidelines,^23^ after the 2017 guidelines update,^24^ and between these two time periods. This approach was aimed at minimizing temporal and selection biases by considering the evolution of the standard of care over time and by retrospectively considering patient subgroups with potentially different survival outcomes. Third, the use of dynamic time‐dependent propensity score matching in the definition of longitudinal analysis was aimed to dampen temporal biases because compared patients were considered within the same four‐month period of the timeline. Matching variables included both time‐dependent and time‐independent variables.

Patients with missing information on vasoactive or vasodilator treatment were excluded from the longitudinal analysis because they were directly involved in the endpoint assessment. Predictive mean matching was used as an imputation method for missed FVC and DLco based on remaining collected predictors and the generation of five imputed data sets. An additional sensitivity analysis based on only completed observations was proposed.

### Minimal sample size definition

It was determined that a minimum of 60 patients in each group would be required to demonstrate a 50% risk reduction (HR = 0.5) in vasoactive or vasodilator treatment escalation with MMF treatment compared with a control group of the same size. This calculation assumed a two‐tailed alpha of 0.05 and a power of 0.9 based on an expected baseline event rate of two events per year in the control group^25^ and an average follow‐up duration of 2.5 years. Additionally, it accounted for an annual censoring rate of 50% in both the intervention and control groups.^26^

The study was conducted within the ethical approval from each participating center according to the SPRING Italian registry policy. The authors confirm that the data supporting the findings of this study are available within the article or its supplementary materials. Patients were not directly involved in setting the research question, the endpoint measures, or the design of this study. However, unmet clinical needs that emerged during routine clinical practice were considered. Patients were informed of the results of this study on request.

## RESULTS

### Patients' characteristics and clinical comparisons of patients receiving and not receiving MMF

The patient selection process is summarized in Supplementary Figure 1. A total of 1,435 patients with lcSSc were enrolled in the SPRING registry as of December 31, 2022. The patients resided throughout the country, particularly in proximity to the main urban centers, where the enrollment sites were located (Supplementary Figure 2A).

The median observation time from the diagnosis of SSc to the last available follow‐up was 105 months (IQR 54–180). MMF was prescribed to 152 (10.6%) patients within the timeframe from diagnosis to the last follow‐up, with a median time from diagnosis to MMF prescription of 28 (IQR 5–82) months. The timeline of MMF prescription is reported in Supplementary Figure 2B.

The baseline clinical characteristics of the overall cohort and the comparison of patients receiving and not receiving MMF before matching are summarized in Table 1. After adjusting for multiple comparisons, patients receiving MMF were more commonly men and presented a higher frequency of a history of ILD and myositis, as well as being anti‐Scl70 positive and ACA negative. The statistical association between MMF prescription with no history of DUs was lost when adjusted for multiple comparisons, although patients with DUs tended to be less likely to have been exposed to MMF compared with patients without any history of these complications due to concomitant organ involvements. Patients who were receiving MMF were also more likely to have been exposed to rituximab. Subgroup analysis confirmed these associations when patients diagnosed before 2007, from 2008 to 2015, and after 2016 were considered in the subgroup analysis, as shown in Supplementary Tables 1 to 3.

**Table 1:** Comparison of clinical variables between patients with lcSSc who were and were not receiving MMF at the last available follow‐up*

	All patients	Patients not receiving MMF	Patients receiving MMF	*P* value
N	1,435	1,283	152	–
Men, n (%)	133 (9.3)	107 (8.3)	26 (17.1)	**<0.001**
Last available CCI, mean ± SD	2.4 ± 1.7	2.4 ± 1.7	2.2 ± 1.5	0.2
Age at SSc onset, mean ± SD, y	49.8 ± 14.2	49.9 ± 14.2	48.7 ± 13.9	0.3
ACA positivity, n (%)	604 (42.1)	589 (45.9)	15 (9.9)	**<0.001**
Anti‐Scl70 positivity, n (%)	395 (27.5)	312 (24.3)	83 (54.6)	**<0.001**
ILD on HRCT, n (%)	573 (39.9)	449 (35.0)	124 (81.6)	**<0.001**
Last available FVC, % of predicted, mean ± SD	103.8 ± 21.8	105.6 ± 20.7	88.8 ± 24.8	**<0.001**
Last available DLco, % of predicted, mean ± SD	69.0 ± 19.4	70.0 ± 18.9	60.8 ± 21.7	**<0.001**
DUs (ever), n (%)	327 (22.8)	303 (23.6)	24 (15.8)	0.030
Myositis, (%)	15 (1.0)	10 (0.8)	5 (3.3)	**0.016**
Baseline CCB, n (%)	961 (67.0)	857 (66.8)	104 (68.4)	0.7
Baseline sildenafil, n (%)	49 (3.4)	42 (3.3)	7 (4.6)	0.4
Baseline tadalafil, n (%)	16 (1.1)	15 (1.2)	1 (0.7)	>0.9
Baseline bosentan, n (%)	329 (22.9)	287 (22.4)	42 (27.6)	0.14
Baseline macitentan, n (%)	32 (2.2)	28 (2.2)	4 (2.6)	0.8
Baseline ambrisentan, n (%)	9 (0.6)	9 (0.7)	0 (0.0)	0.6
Baseline iloprost, n (%)	718 (50.0)	646 (50.4)	72 (47.4)	0.5
Baseline rituximab, n (%)	33 (2.3)	25 (1.9)	8 (5.3)	**0.018**
Baseline tocilizumab, n (%)	28 (2.0)	25 (1.9)	3 (2.0)	**>0**.**9**

* Bold *P* values indicate statistically significant differences with the threshold set at 0.0025 after Bonferroni adjustment for multiple comparisons. ACA, anti‐centromere antibody; CCB, calcium channel blocker; CCI, Charlson Comorbidity Index; DU, digital ulcer; DLco, diffusing capacity of the lung for carbon monoxide; FVC, forced vital capacity; HRCT, high‐resolution computed tomography; ILD, interstitial lung disease; IQR, interquartile range; lcSSc, limited cutaneous systemic sclerosis; MMF, mycophenolate mofetil.

### MMF treatment and vasoactive or vasodilator treatment escalation

**Figure 2:**
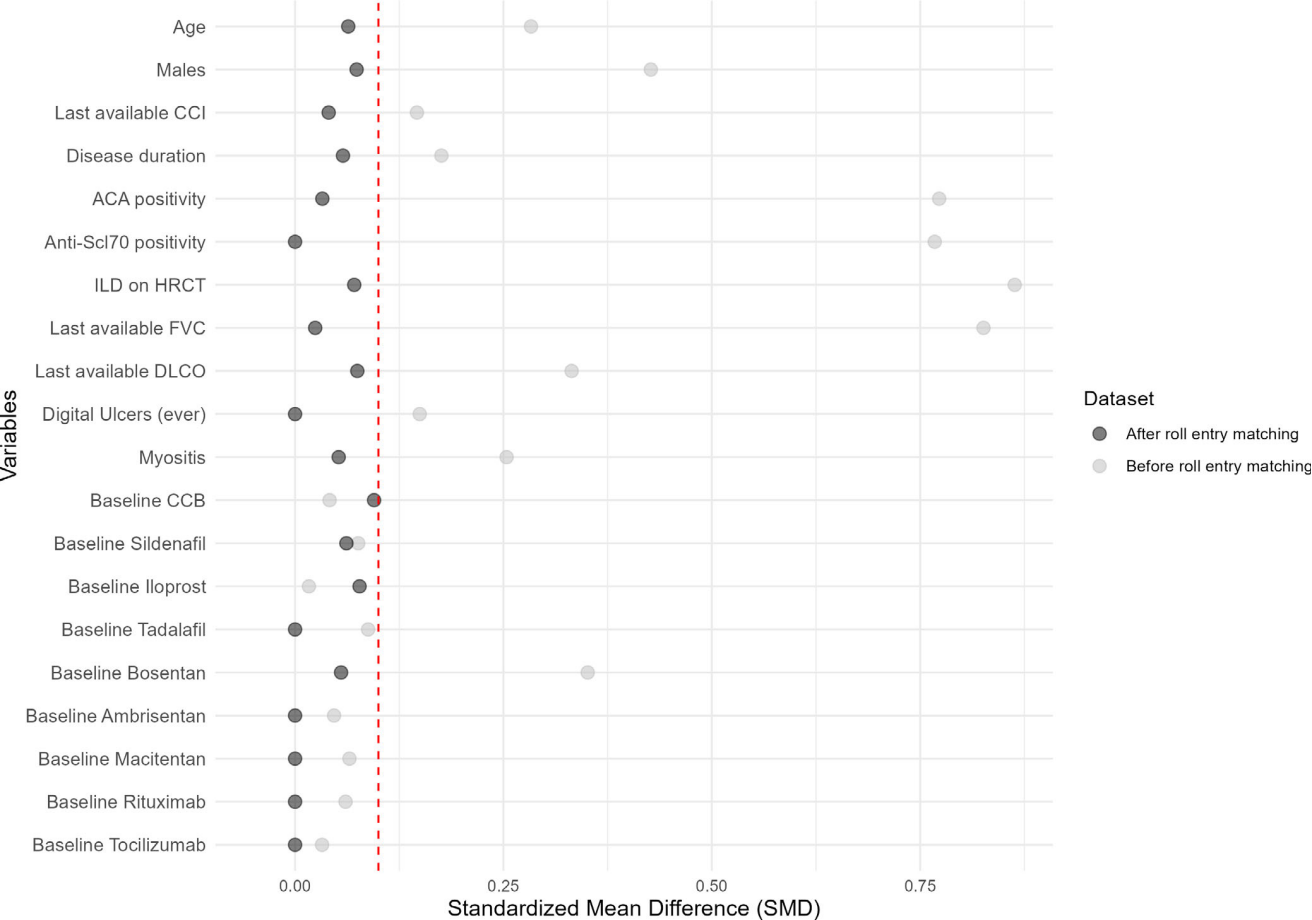
Love plot showing balancing covariates before and after roll‐entry matching. ACA, anti‐centromere antibody; CCB, calcium channel blocker; CCI, Charlson Comorbidity Index; DLco, diffusing capacity of the lung for carbon monoxide; FVC, forced vital capacity; HRCT, high‐resolution computed tomography; ILD, interstitial lung disease. Color figure can be viewed in the online issue, which is available at http://onlinelibrary.wiley.com/doi/10.1002/acr.70039/abstract.

Out of 152 patients who received MMF, 19 discontinued the medication within the first three months, and 26 were excluded because of incomplete information in follow‐up. Consequently, 107 patients remained and were matched with 107 controls who were unexposed to MMF using a time‐dependent propensity score. This matching was deemed successful based on SMD values under 0.1, as summarized in Figure 2 and detailed in Table 2. In a quasi‐experimental design, a total of 214 patients were followed for a median duration of 40.5 months (IQR 23.3–60.0) after the index date, which was the date of MMF initiation in the intervention group and the date of matching for the control group.

**Table 2:** Comparison of clinical variables between patients with lcSSc who were and were not prescribed MMF during follow‐up after roll‐entry matching*

	Patients not prescribed MMF	Patients prescribed MMF	SMD
N	107	107	–
Men, n (%)	20 (18.7)	17 (15.9)	0.074
Age, mean ± SD	53.78 ± 14.18	54.64 ± 12.74	0.064
Last available CCI, mean ± SD	2.31 ± 1.64	2.24 ± 1.61	0.040
Disease duration, mean ± SD	5.49 ± 5.01	5.83 ± 6.88	0.057
ACA positivity, n (%)	10 (9.3)	9 (8.4)	0.033
Anti‐Scl70 positivity, n (%)	65 (60.7)	65 (60.7)	<0.001
ILD on HRCT, n (%)	85 (79.4)	88 (82.2)	0.071
Last available FVC, % of predicted, mean ± SD	85.57 ± 18.95	89.03 ± 25.49	0.024
Last available DLco, % of predicted, mean ± SD	62.14 ± 17.70	60.64 ± 22.12	0.075
DUs (ever), n (%)	21 (19.6)	21 (19.6)	<0.001
Myositis, n (%)	4 (3.7)	3 (2.8)	0.053
Baseline CCB, n (%)	46 (43.0)	41 (38.3)	0.095
Baseline sildenafil, n (%)	3 (2.8)	2 (1.9)	0.062
Baseline tadalafil, n (%)	0 (0.0)	0 (0.0)	<0.001
Baseline bosentan, n (%)	15 (14.0)	13 (12.1)	0.055
Baseline ambrisentan, n (%)	0 (0.0)	0 (0.0)	<0.001
Baseline macitentan, n (%)	0 (0.0)	0 (0.0)	<0.001
Baseline iloprost, n (%)	41 (38.3)	37 (34.6)	0.078
Baseline rituximab, n (%)	0 (0.0)	0 (0.0)	<0.001
Baseline tocilizumab, n (%)	0 (0.0)	0 (0.0)	<0.001

* ACA, anti‐centromere antibody; CCB, calcium channel blocker; CCI, Charlson Comorbidity Index; DU, digital ulcer; DLco, diffusing capacity of the lung for carbon monoxide; FVC, forced vital capacity; HRCT, high‐resolution computed tomography; ILD, interstitial lung disease; IQR, interquartile range; lcSSc, limited cutaneous systemic sclerosis; MMF, mycophenolate mofetil; SMD, standardized mean difference.

**Figure 3:**
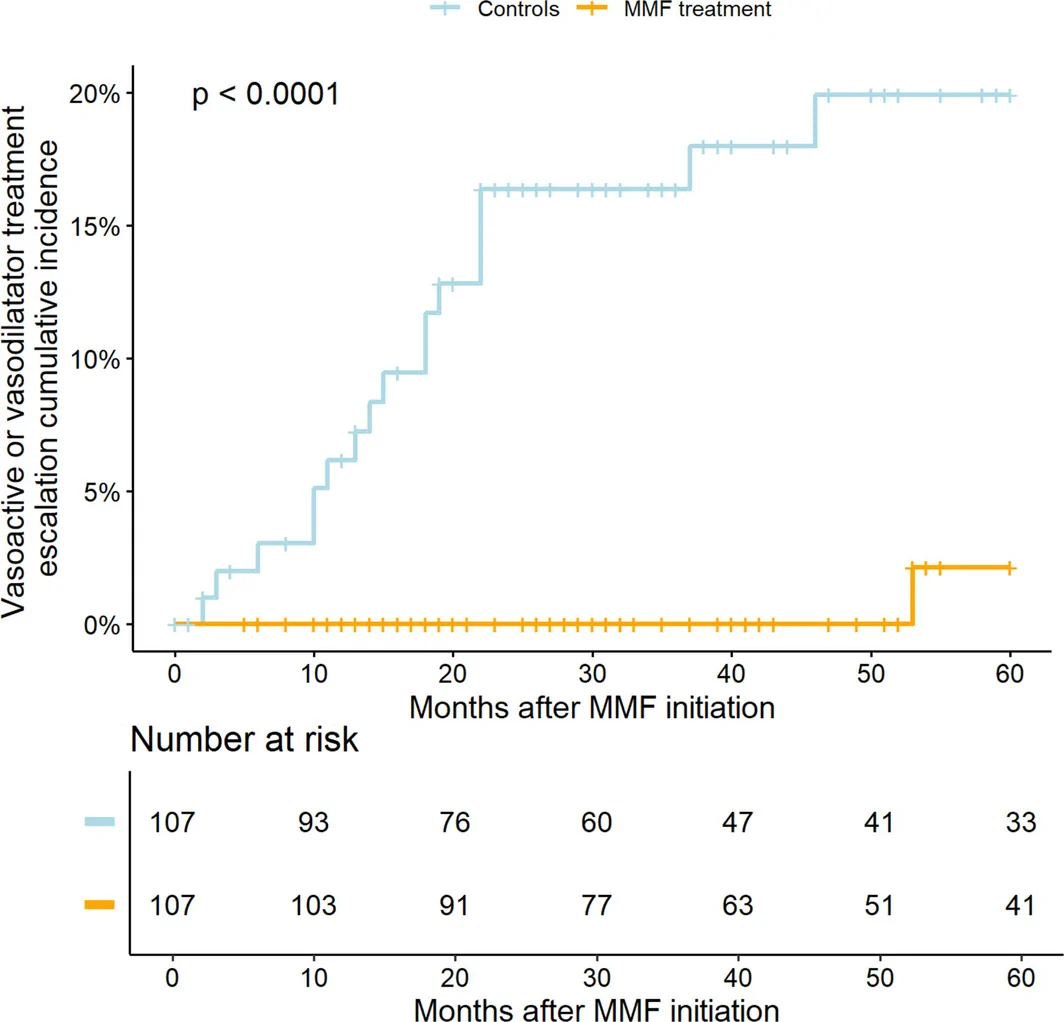
Comparison of cumulative incidence curves of vasoactive or vasodilator treatment escalation in MMF‐treated patients and matched controls. MMF, mycophenolate mofetil.

During follow‐up, 18 instances of escalated vasoactive or vasodilator treatment were recorded. This corresponds to an incidence rate of 2.2 events per 1,000 patient‐years. Escalation of treatment was due to uncontrolled DUs in 15 patients and severe Raynaud phenomenon in 3 patients. Treatment initiation included iloprost initiation in 11 patients, a combination of bosentan and iloprost initiation in 6 patients, and bosentan initiation in 1 patient (Supplementary Table 4). One single event was reported in the MMF‐treated group (0.3 per 100 patient‐years), whereas 17 occurred in the control group (5.4 per 100 patient‐years). At the end of follow‐up, the cumulative incidence of events was 2.5% (95% CI 0.0–7.2) in the MMF group versus 21.8% (95% CI 10.0–32.1) in the control group. The survival distributions between the two groups were significantly different, as indicated by the log‐rank test (*P* value = 0.00025) (Figure 3). Univariate Cox regression analysis revealed that MMF treatment was associated with a 95% reduction in risk (HR 0.05, 95% CI 0.01–0.38; *P* value = 0.004) of vascular complications requiring escalation of vasoactive/vasodilator therapy when compared with the matched control group with comparable clinical characteristics.

In the sensitivity analysis, which included only 86 patients with complete clinical observations for each group, the estimated cumulative incidence of vasoactive or vasodilator treatment was 1.2% (IQR 0.0%–7.3%) in the MMF group and 22.8% (10.0%–33.1%) in the control group. A statistically significant difference was confirmed (*P* value <0.001), corresponding to a HR of 0.06 (95% CI 0.01–0.47; *P* value = 0.007) in the Cox analysis (Supplementary Figure 3).

## DISCUSSION

Our results show that in the last decades patients with lcSSc were more likely to receive MMF, although in our retrospective cohort only 1 of 10 received MMF. Most importantly, our data suggest that MMF exerts a protective effect against vascular complications. In our cohort, the main clinical characteristic associated with MMF treatment appeared to be ILD, followed by the occurrence of myositis. Consistently, ILD‐related risk factors, such as male sex, anti‐Scl70 positivity, and ACA negativity, were more frequently observed in the MMF receiving group. Interestingly, neither comorbidity burden nor age seemed to influence the prescription of MMF. Additionally, we investigated whether there was any added benefit in preventing the need for a vascular or vasoactive treatment upgrade, wich served as a surrogate event for the onset of uncontrolled vascular disease. Using a propensity score matching strategy, which is a commonly used strategy to simulate randomization in observational studies, we defined a control group. Traditionally, propensity score matching designs are cross‐sectional, matching on covariates before the intervention and measuring endpoints after the intervention to analyze the effect of treatment at a specific point in time. Although effective in many situations, this approach assumes that covariates do not change in a relevant time window, or if they do, that these changes will not affect the endpoint variable. Additionally, identifying the index date for the unexposed control group can introduce substantial biases. Time‐dependent propensity score matching, associated with a rolling entry strategy, overcomes these limitations and moves closer to a classical randomized study design.

The results of this comparison, corroborated by the sensitivity analysis, indicate that MMF significantly decreases the risk of upgrading to vasoactive or vasodilator treatment. The recorded events were attributed to acral vascular complications. This is not surprising because MMF tended to be initiated early after diagnosis, resulting in shorter disease duration among control group patients, as well. Moreover, because the matching strategy favored patients with similar characteristics, those on macitentan, ambrisentan, tadalafil, and sildenafil, representing a minority group with PAH, were more difficult to match and therefore were less represented in the selection process.

This finding is fully consistent with both preclinical studies reporting the protective effect of MMF on endothelial activation and intimal proliferation in both in vitro and animal models,^5^, ^6^, ^7^, ^8^, ^9^ as well as with the beneficial effect reported in patients who received MMF to prevent solid organ rejection.^8^, ^10^, ^11^ Moreover, these data support a more unified vision of SSc pathophysiology in which, other than vasoactive and vasodilators therapy, dampening the immune response could counteract microvascular damage underlying SSc.

The MINIMISE randomized controlled trial is currently evaluating the effects of MMF in a lcSSc population with no clinical recommended indication for an immunosuppressive treatment (exclusion criteria are the presence of lung or cardiac involvement) (EudraCT: 2019‐004139‐21). It will be interesting to see if in this population MMF will also have protective effect on the vascular compartment because in our study the patients with lcSSc who underwent MMF were those with the presence of organ involvement (ie, characterized by a significant pro‐inflammatory and profibrotic burden). Therefore, we cannot exclude the possibility that the effect of MMF on the microvascular compartment was not direct, but rather mediated through the control of fibrotic and immune burden.

Some limitations should be acknowledged. First, propensity score matching cannot account for unmeasured or unknown confounding variables, which potentially bias the estimated treatment effect. Second, the retrospective design may have affected the representativeness of the population examined. Specifically, only long‐term survivors diagnosed earlier in the evaluated timeline were included, which may limit the generalizability of the findings. Finally, smoke exposure and the specific MMF dose were not available. Similarly, the specific predominant reason for MMF introduction (eg, ILD, myositis, myocarditis, and early disease with risk factors for the development of a diffuse form) could not be retrospectively reconstructed.

In conclusion, our retrospective analysis indicated that, during the study period, MMF treatment in lcSSc was limited to patients with risk factors and visceral or musculoskeletal involvement approaching the severity characteristic of dcSSc. In our study, MMF treatment was associated with a reduced need for vasoactive or vasodilative therapy escalation, suggesting a beneficial effect on SSc microvascular complications. Randomized controlled studies are warranted to confirm the effects of MMF on vascular SSc manifestations.

## AUTHOR CONTRIBUTIONS

All authors contributed to at least one of the following manuscript preparation roles: conceptualization AND/OR methodology, software, investigation, formal analysis, data curation, visualization, and validation AND drafting or reviewing/editing the final draft. As corresponding author, Dr Natalello confirms that all authors have provided the final approval of the version to be published and takes responsibility for the affirmations regarding article submission (eg, not under consideration by another journal), the integrity of the data presented, and the statements regarding compliance with institutional review board/Declaration of Helsinki requirements.

## Supporting information


**Disclosure form**.

**Supplementary Figure 1:** Patient selection process flow‐chart. Abbreviations: dcSSc (diffuse cutaneous Systemic Sclerosis), lcSSc (limited cutaneous Systemic Sclerosis), MMF (Mycophenolate Mofetil), SIR (Italian Society of Rheumatology), VEDOSS (Very Early Diagnosis of Systemic Sclerosis).
**Supplementary Figure 2:** Geographical distribution of the 1435 lcSSc patients enrolled in the study (A) and temporal distribution of drug initiation in MMF‐treated patients (B). The black dots indicate the sites. Abbreviations: lcSSc (limited cutaneous Systemic Sclerosis), MMF (Mycophenolate Mofetil).
**Supplementary Figure 3:** Comparison of cumulative incidence curves of vasoactive or vasodilator treatment escalation in MMF‐treated patients and matched controls, including patients without missing data only. Abbreviations: MMF (Mycophenolate Mofetil).
**Supplementary Table 1:** Comparison of clinical variables between patients with lcSSc diagnosed from 1980 to 2007 who were and were not prescribed MMF at the last available follow‐up.
**Supplementary Table 2:** Comparison of clinical variables between patients with lcSSc diagnosed from 2008 to 2015 who were and were not prescribed MMF at the last available follow‐up.
**Supplementary Table 3:** Comparison of clinical variables between patients with lcSSc diagnosed from 2016 to 2022 who were and were not prescribed MMF at the last available follow‐up.
**Supplementary Table 4:** Characterization of the vasoactive or vasodilator treatment escalation events.
