# Supplementary material for: Mycophenolate Mofetil Treatment Reduces the Risk of Treatment Escalation Due to Vascular Complications in Limited Cutaneous Systemic Sclerosis: Emulation of a Target Trial From the Italian Rheumatology Society SPRING Registry
Source: Arthritis Care Res (Hoboken). 2026 Apr 17;78(9):1130–9. doi: 10.1002/acr.70039 (PMC13511392; doi:10.1002/acr.70039)
Supplement: Supplementary file 2 [file ACR-78-1130-s002.docx]

**MYCOPHENOLATE MOFETIL TREATMENT REDUCES THE RISK OF TREATMENT ESCALATION DUE TO VASCULAR COMPLICATIONS IN LIMITED CUTANEOUS SYSTEMIC SCLEROSIS: EMULATION OF A TARGET TRIAL FROM ITALIAN SIR-SPRING REGISTRY**

De Lorenzis E et al.

**SUPPLEMENTARY MATERIAL**

**Supplementary Figure 1: Patient selection process flow-chart.** Abbreviations: dcSSc (diffuse cutaneous Systemic Sclerosis), lcSSc (limited cutaneous Systemic Sclerosis), MMF (Mycophenolate Mofetil), SIR (Italian Society of Rheumatology), VEDOSS (Very Early Diagnosis of Systemic Sclerosis).

**Supplementary Figure 2: Geographical distribution of the 1435 lcSSc patients enrolled in the study (A) and temporal distribution of drug initiation in MMF-treated patients (B).** The black dots indicate the sites. Abbreviations: lcSSc (limited cutaneous Systemic Sclerosis), MMF (Mycophenolate Mofetil).

**Supplementary Figure 3: Comparison of cumulative incidence curves of vasoactive or vasodilator treatment escalation in MMF-treated patients and matched controls, including patients without missing data only.** Abbreviations: MMF (Mycophenolate Mofetil).

**Supplementary Table 1: Comparison of clinical variables between patients with lcSSc diagnosed from 1980 to 2007 who were and were not prescribed MMF at the last available follow-up.**

| **LcSSc patients 1980-2007** | **All patients** | **Patients not prescribed MMF** | **Patients prescribed MMF** | **p-value** |
| --- | --- | --- | --- | --- |
| **N** | 558 | 518 | 40 | - |
| **Males, n (%)** | 41 (7·3%) | 35 (6·8%) | 6 (15·0%) | 0·10 |
| **Last available CCI, mean±SD** | 2·2±1·6 | 2·2±1·6 | 1·9±1·5 | 0·2 |
| **Age at SSc onset, years, mean±SD** | 45·0±13·0 | 45·4±12·8 | 39·7±14·6 | **0·008** |
| **ACA positivity, n (%)** | 256 (45·9%) | 250 (48·3%) | 6 (15·0%) | **<0·001** |
| **Anti-Scl70 positivity, n (%)** | 138 (24·7%) | 119 (23·0%) | 19 (47·5%) | **<0·001** |
| **ILD on HRCT, n (%)** | 241 (43·2%) | 209 (40·3%) | 32 (80·0%) | **<0·001** |
| **Last available FVC, % of predicted, mean±SD** | 104·3±22·1 | 105·9±21·0 | 84·6±26·5 | **<0·001** |
| **Last available DLCO, % of predicted, mean±SD** | 66·2±18·6 | 66·7±18·1 | 59·8±23·7 | **0·023** |
| **Digital Ulcers (ever), n (%)** | 173 (31·0%) | 165 (31·9%) | 8 (20·0%) | 0·12 |
| **Myositis, (%)** | 2 (0·4%) | 1 (0·2%) | 1 (2·5%) | 0·14 |
| **Baseline CCB, n (%)** | 375 (67·2%) | 349 (67·4%) | 26 (65·0%) | 0·8 |
| **Baseline Sildenafil, n (%)** | 27 (4·8%) | 26 (5·0%) | 1 (2·5%) | 0·7 |
| **Baseline Tadalafil, n (%)** | 8 (1·4%) | 8 (1·5%) | 0 (0·0%) | >0·9 |
| **Baseline Bosentan, n (%)** | 170 (30·5%) | 154 (29·7%) | 16 (40·0%) | 0·2 |
| **Baseline Macitentan, n (%)** | 17 (3·0%) | 16 (3·1%) | 1 (2·5%) | >0·9 |
| **Baseline Ambrisentan, n (%)** | 6 (1·1%) | 6 (1·2%) | 0 (0·0%) | >0·9 |
| **Baseline Iloprost, n (%)** | 329 (59·0%) | 305 (58·9%) | 24 (60·0%) | 0·9 |
| **Baseline Rituximab, n (%)** | 16 (2·9%) | 15 (2·9%) | 1 (2·5%) | >0·9 |
| **Baseline Tocilizumab, n (%)** | 11 (2·0%) | 10 (1·9%) | 1 (2·5%) | >0·9 |

*Bold p-values indicate statistically significant differences with the threshold set at 0·0025 after Bonferroni adjustment for multiple comparisons. Abbreviations: ACA (Anti-Centromere Antibody), CCB (Calcium Channel Blocker), CCI (Charlson Comorbidity Index), DLCO (Diffusing Capacity of the Lung for Carbon Monoxide), FVC (Forced Vital Capacity), HRCT (High-Resolution Computed Tomography), ILD (Interstitial Lung Disease), IQR (Interquartile Range), lcSSc (limited cutaneous Systemic Sclerosis), MMF (Mycophenolate Mofetil), SD (Standard Deviation).*

**Supplementary Table 2: Comparison of clinical variables between patients with lcSSc diagnosed from 2008 to 2015 who were and were not prescribed MMF at the last available follow-up.**

| **LcSSc patients 2008-2015** | **All patients** | **Patients not prescribed MMF** | **Patients prescribed MMF** | **p-value** |
| --- | --- | --- | --- | --- |
| **N** | 678 | 593 | 85 |  |
| **Males, n (%)** | 61 (9·0%) | 47 (7·9%) | 14 (16·5%) | 0·010 |
| **Last available CCI, mean±SD** | 2·6±1·7 | 2·6±1·7 | 2·6±1·7 | **0·4** |
| **Age at SSc onset, years, mean±SD** | 52·9±14·1 | 52·9±14·3 | 52·4±12·5 | 0·8 |
| **ACA positivity, n (%)** | 264 (38·9%) | 258 (43·5%) | 6 (7·1%) | **<0·001** |
| **Anti-Scl70 positivity, n (%)** | 198 (29·2%) | 148 (25·0%) | 50 (58·8%) | **<0·001** |
| **ILD on HRCT, n (%)** | 260 (38·3%) | 190 (32·0%) | 70 (82·4%) | **<0·001** |
| **Last available FVC, % of predicted, mean±SD** | 103·2±21·0 | 105·4±19·9 | 88·5±22·4 | **<0·001** |
| **Last available DLCO, % of predicted, mean±SD** | 69·9±19·8 | 71·2±19·1 | 61·1±22·3 | **<0·001** |
| **Digital Ulcers (ever), n (%)** | 118 (17·4%) | 105 (17·7%) | 13 (15·3%) | 0·6 |
| **Myositis, (%)** | 9 (1·3%) | 5 (0·8%) | 4 (4·7%) | 0·018 |
| **Baseline CCB, n (%)** | 450 (66·4%) | 391 (65·9%) | 59 (69·4%) | 0·5 |
| **Baseline Sildenafil, n (%)** | 18 (2·7%) | 13 (2·2%) | 5 (5·9%) | 0·063 |
| **Baseline Tadalafil, n (%)** | 7 (1·0%) | 6 (1·0%) | 1 (1·2%) | >0·9 |
| **Baseline Bosentan, n (%)** | 147 (21·7%) | 123 (20·7%) | 24 (28·2%) | 0·12 |
| **Baseline Macitentan, n (%)** | 14 (2·1%) | 11 (1·9%) | 3 (3·5%) | 0·4 |
| **Baseline Ambrisentan, n (%)** | 3 (0·4%) | 3 (0·5%) | 0 (0·0%) | >0·9 |
| **Baseline Iloprost, n (%)** | 333 (49·1%) | 295 (49·7%) | 38 (44·7%) | 0·4 |
| **Baseline Rituximab, n (%)** | 17 (2·5%) | 10 (1·7%) | 7 (8·2%) | 0·003 |
| **Baseline Tocilizumab, n (%)** | 17 (2·5%) | 15 (2·5%) | 2 (2·4%) | >0·9 |

*Bold p-values indicate statistically significant differences with the threshold set at 0·0025 after Bonferroni adjustment for multiple comparisons· Abbreviations: ACA (Anti-Centromere Antibody), CCB (Calcium Channel Blocker), CCI (Charlson Comorbidity Index), DLCO (Diffusing Capacity of the Lung for Carbon Monoxide), FVC (Forced Vital Capacity), HRCT (High-Resolution Computed Tomography), ILD (Interstitial Lung Disease), IQR (Interquartile Range), lcSSc (limited cutaneous Systemic Sclerosis), MMF (Mycophenolate Mofetil), SD (Standard Deviation).*

**Supplementary Table 3: Comparison of clinical variables between patients with lcSSc diagnosed from 2016 to 2022 who were and were not prescribed MMF at the last available follow-up.**

| **LcSSc patients 2016-2022** | **All patients** | **Patients not prescribed MMF** | **Patients prescribed MMF** | **p-value** |
| --- | --- | --- | --- | --- |
| **N** | 199 | 172 | 27 | - |
| **Males, n (%)** | 31 (15·6%) | 25 (14·5%) | 6 (22·2%) | 0·4 |
| **Last available CCI, mean±SD** | 2·5±1·8 | 2·5±1·8 | 2·2±1·5 | 0·3 |
| **Age at SSc onset, years, mean±SD** | 52·7±14·1 | 53·0±14·5 | 50·3±11·5 | 0·4 |
| **ACA positivity, n (%)** | 84 (42·2%) | 81 (47·1%) | 3 (11·1%) | **<0·001** |
| **Anti-Scl70 positivity, n (%)** | 59 (29·6%) | 45 (26·2%) | 14 (51·9%) | **0·007** |
| **ILD on HRCT, n (%)** | 72 (36·2%) | 50 (29·1%) | 22 (81·5%) | **<0·001** |
| **Last available FVC, % of predicted, mean±SD** | 104·9±22·3 | 106·9±20·2 | 91·9±30·2 | **0·001** |
| **Last available DLCO, % of predicted, mean±SD** | 73·8±20·0 | 74·6±19·6 | 68·6±21·8 | 0·15 |
| **Digital Ulcers (ever), n (%)** | 36 (18·1%) | 33 (19·2%) | 3 (11·1%) | 0·4 |
| **Myositis, (%)** | 4 (2·0%) | 4 (2·3%) | 0 (0·0%) | >0·9 |
| **Baseline CCB, n (%)** | 136 (68·3%) | 117 (68·0%) | 19 (70·4%) | 0·8 |
| **Baseline Sildenafil, n (%)** | 4 (2·0%) | 3 (1·7%) | 1 (3·7%) | 0·4 |
| **Baseline Tadalafil, n (%)** | 1 (0·5%) | 1 (0·6%) | 0 (0·0%) | >0·9 |
| **Baseline Bosentan, n (%)** | 56 (28·1%) | 46 (26·7%) | 10 (37·0%) | 0·3 |
| **Baseline Macitentan, n (%)** | 12 (6·0%) | 10 (5·8%) | 2 (7·4%) | 0·7 |
| **Baseline Ambrisentan, n (%)** | 1 (0·5%) | 1 (0·6%) | 0 (0·0%) | >0·9 |
| **Baseline Iloprost, n (%)** | 0 (0·0%) | 0 (0·0%) | 0 (0·0%) | - |
| **Baseline Rituximab, n (%)** | 0 (0·0%) | 0 (0·0%) | 0 (0·0%) | - |
| **Baseline Tocilizumab, n (%)** | 0 (0·0%) | 0 (0·0%) | 0 (0·0%) | - |

*Bold p-values indicate statistically significant differences with the threshold set at 0·0025 after Bonferroni adjustment for multiple comparisons· Abbreviations: ACA (Anti-Centromere Antibody), CCB (Calcium Channel Blocker), CCI (Charlson Comorbidity Index), DLCO (Diffusing Capacity of the Lung for Carbon Monoxide), FVC (Forced Vital Capacity), HRCT (High-Resolution Computed Tomography), ILD (Interstitial Lung Disease), IQR (Interquartile Range), lcSSc (limited cutaneous Systemic Sclerosis), MMF (Mycophenolate Mofetil), SD (Standard Deviation).*

**Supplementary Table 4: Characterization of the vasoactive or vasodilator treatment escalation events.**

|  | **Gender** | **Age** | **Group** | **Time** | **Treatment upgrade** | **Indication** |
| --- | --- | --- | --- | --- | --- | --- |
| 1 | Female | 42 | Control | 11-2005 | Bosentan + Iloprost | DUs |
| 2 | Female | 50 | MMF | 07-2008 | Bosentan | DUs |
| 3 | Male | 50 | Control | 11-2009 | Bosentan + Iloprost | DUs |
| 4 | Male | 61 | Control | 10-2010 | Bosentan + Iloprost | DUs |
| 5 | Female | 21 | Control | 03-2011 | Iloprost | DUs |
| 6 | Male | 47 | Control | 11-2012 | Iloprost | DUs |
| 7 | Female | 74 | Control | 07-2013 | Bosentan + Iloprost | DUs |
| 8 | Female | 24 | Control | 11-2013 | Iloprost | Severe RP |
| 9 | Male | 19 | Control | 11-2013 | Bosentan + Iloprost | DUs |
| 10 | Female | 55 | Control | 03-2014 | Iloprost | DUs |
| 11 | Female | 65 | Control | 03-2014 | Iloprost | DUs |
| 12 | Female | 36 | Control | 11-2014 | Iloprost | DUs |
| 13 | Female | 43 | Control | 10-2015 | Iloprost | Severe RP |
| 14 | Male | 65 | Control | 03-2016 | Iloprost | Severe RP |
| 15 | Male | 69 | Control | 11-2016 | Bosentan + Iloprost | DUs |
| 16 | Female | 46 | Control | 01-2017 | Iloprost | DUs |
| 17 | Female | 51 | Control | 07-2021 | Iloprost | DUs |
| 18 | Female | 32 | Control | 09-2021 | Iloprost | DUs |

*Abbreviations: DU (Digital Ulcer), MMF (Mycophenolate Mofetil), RP (Raynaud’s phenomenon).*
